# DIGITAL SKILLS, STEM OCCUPATION, AND JOB AUTOMATION RISK AMONG THE OLDER WORKERS IN THE US

**DOI:** 10.1093/geroni/igad104.2158

**Published:** 2023-12-21

**Authors:** Takashi Yamashita, Donnette Narine, Runcie Chidebe, Phyllis Cummins, Jenna Kramer, Rita Karam

**Affiliations:** University of Maryland, Baltimore County, Baltimore, Maryland, United States; University of Maryland Baltimore, Baltimore, Maryland, United States; Miami University, Oxford, Ohio, United States; Miami University, Oxford, Ohio, United States; RAND Corporation, Arlington, Virginia, United States; RAND Corporation, Santa Monica, California, United States

## Abstract

Rapidly advancing technologies are replacing human labor. Particularly, occupations with more routine tasks, such as sales and simple food preparations, are known to be at a higher risk of job automation than those with non-routine tasks, such as STEM occupations (e.g., engineering, health care). Additionally, an aging workforce generally faces higher job automation risks due to skill obsolescence and lower digital skills than younger counterparts. A better understanding of the associations between job automation risk, digital skills, and STEM (vs. non-STEM) occupations can facilitate preparations for continuing job automation and population aging. We analyzed a nationally representative sample of middle-aged and older U.S. workers aged 50 to 74 (n = 1,570) from the 2012/2014/2017 Program for International Assessment of Adult Competencies (PIAAC) restricted-use-file data. The estimated job automation risks (i.e., % of jobs to be automated in the next decades) by occupation were derived from the previous studies. PIAAC digital problem-solving skills proficiency (0-500 points) was assessed based on a series of practical digital tasks (e.g., sorting emails by responses in an application). Results of the survey-weighted linear regression showed that the greater digital skill proficiency (b = -0.04, p < 0.05) and STEM occupations (b = -17.18, p < 0.05) are associated with lower job automation risks, even after adjusting for a series of demographic, socioeconomic and civic engagement characteristics. Education and labor policy interventions to promote digital skills among older workers and non-STEM workers may better prepare the aging workforce for the future labor market in the U.S.

